# Ambassadors of peace: The anthropology of war and how to overcome the human killing instinct

**DOI:** 10.1038/s44319-024-00231-5

**Published:** 2024-08-19

**Authors:** Gian Paolo Dotto

**Affiliations:** 1 Cutaneous Biology Research Center, Massachusetts General Hospital and Harvard Medical School, Boston, MA USA; 2 Personalized Cancer Prevention Program, Lausanne University Hospital, Lausanne, Switzerland; 3 International Cancer Prevention Institute, Epalinges, Switzerland

**Keywords:** Evolution & Ecology, History & Philosophy of Science

## Abstract

Understanding the biological and evolutionary factors that make humans go to war may help to overcome our innate tendencies to kill each other.

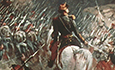

Disease is disorder, a loss of homeostasis, and, if it is left untreated, can destroy the body. Diseases not only affect individuals, but also societies and they do not necessarily need biological causes: stress and traumatic experiences can cause mental disorders as destructive and devastating as infections or cancer. Taking this line of argument further, one might argue that war is an endemic disease of human societies that makes human groups pick up arms and kill their neighbors.

… “one might argue that war is an endemic disease of human societies that makes human groups pick up arms and kill their neighbors.”

A prerequisite for curing a disease is to understand its causes. It is also crucial for a physician to consider the specific context of their patients to inform them about their condition, its symptoms and effects, and ensure their collaboration and compliance with the prescribed treatment. Biomedical research and education are thus called in grave moments to heal individuals as well as societies, coming to the rescue whenever mankind is suffering from disease, whether it is an infection, cancer, a pandemic—or war (Fig. 1).

**Figure 1:**
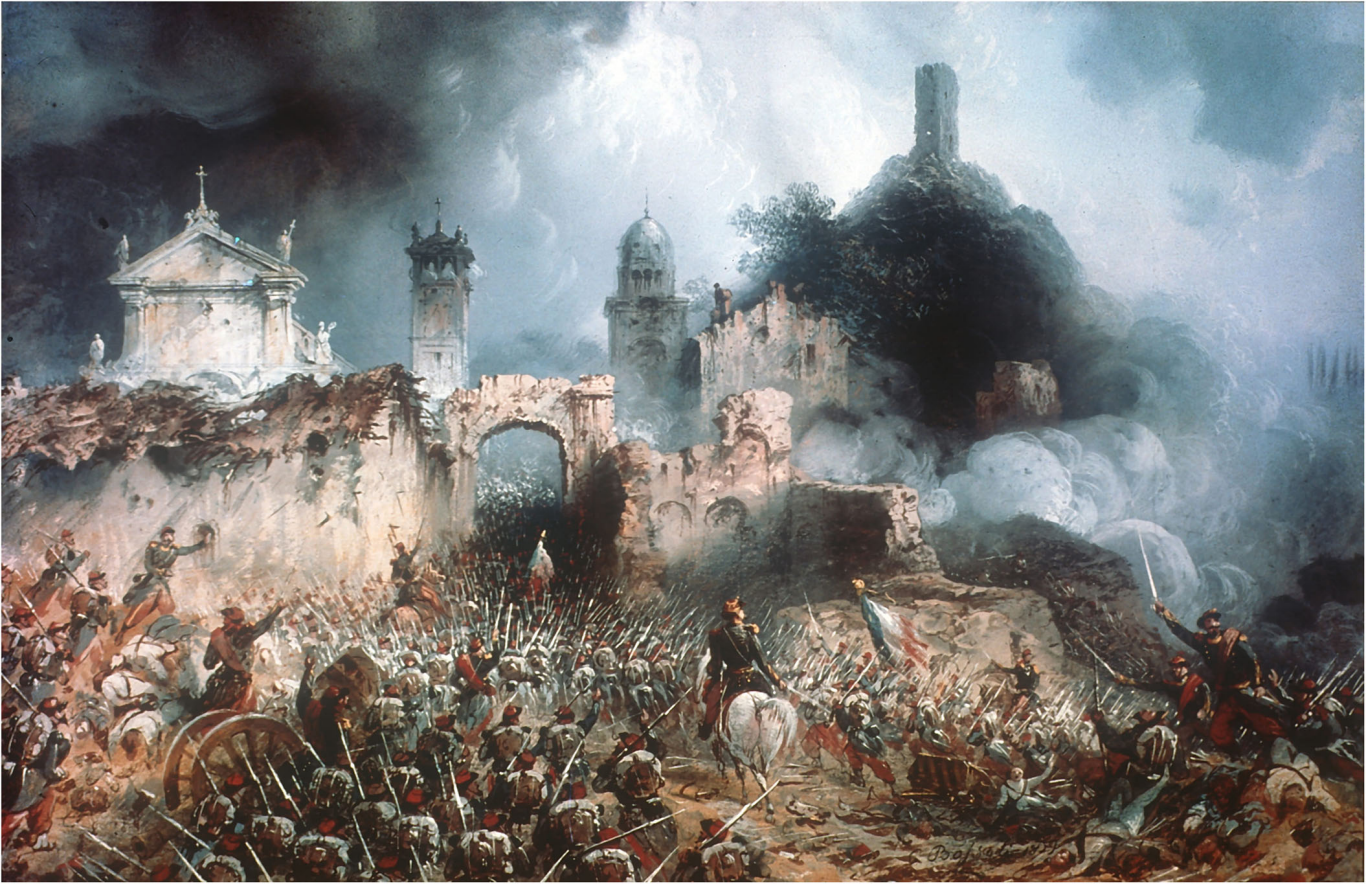
Battle of Solferino (1859), Litography by Carlo Bossoli. Wikimedia/Public Domain. The battle was fought on June 24, 1859 during the second Italian War of Independence and ended with the victory of the allied French and Piedmontese-Sardinian armies against the Austrian army. More than 5000 soldiers were killed and more than 30,000 were wounded or went missing. Henri Dunant who witnessed the aftermath of the battle was so horrified by the carnage and the fate of the wounded left untreated on the battlefield that he started a campaign that would eventually lead to the establishment of the Red Cross and the Geneva Conventions.

In light of the ongoing wars and armed conflicts in Europe, the Middle East and elsewhere that threaten to spread further, considerations about the causes of war and how to prevent it are increasingly important. While the immediate reasons that triggered these conflicts may be attributed to a few individuals or groups, their true causes are often deeply rooted in history. Nonetheless, attempting to explain the current situation by tracing events back 50-100 years or even further disregards more basic factors related to human biology and societal organization (https://www.britannica.com/topic/war/The-causes-of-war). In this context, it is worth exploring the anthropology of warfare (Glowacki et al, 2020) and evolved tendencies to kill that are inherent to *Homo sapiens* (Daly and Wilson, 1988).

While war and peace are intertwined realities that evolved along with ever more sophisticated human societies, the “deep versus shallow roots of war”—whether it was already prevalent among hunter-gatherer tribes or emerged with agriculture which enabled larger, more organized societies—remains a matter of considerable debate (Meijer, 2024). A related and essential question is whether it is possible to create social systems that are permanently free of war, based on mutual dependence, shared values and symbols that incentivize peace instead of aggression and social institutions that discourage and sanction violence (Fry, 2012). These topics, often overshadowed by the overwhelming number of opinion articles on the historical, economic or political causes of contemporary armed conflicts, hold significance beyond academic interest and merit more attention in public discourse.

## Us and the chimpanzees

There is a prevailing assumption that the widespread popular support for declaring war and supporting its continuation despite deaths and hardships stems from extensive propaganda and disinformation campaigns. If the factual truths were known, “common” people, who are intrinsically good, would disapprove of the aggression and crimes committed in their names. In times of war, however, a profound and unsurmountable division arises between “us” and “them”, between the “good” and the “bad”, so that ethical questions concerning the displacement, grave suffering and death of those on the opposing side tend to be ignored or discounted. Warring factions forget that they often have common genetic and ancestral roots, and the ensuing fratricidal conflicts are hard to resolve.

“Warring factions forget that they often have common genetic and ancestral roots, and the ensuing fratricidal conflicts are hard to resolve.”

To place these issues in a broader, biological context, we need to look at our next relatives, the chimpanzees. Since Jane Goodall’s groundbreaking research on their behaviour, many studies have documented violence, murderous acts and brutal raids among rival chimpanzee groups. These primates engage in preferential and planned aggression targeting weaker and isolated members of other groups or even within their own group to improve their dominance and access to resources. Such behavior suggests the presence of evolutionary forces that evolved and maintain a “killer instinct”, at least among primates and humans (Raine and Yang, 2006), and has inspired much research on the anthropology of war to understand its human equivalent.

Mutual killing among chimpanzees or humans can occur within the same group or between different groups. A major difference between us and chimpanzees is that most humans live in highly organized societies that discourage and sanction intragroup violence, whereas chimpanzees live in fission–fusion societies where both intragroup and intergroup aggression occur for competition of resources or mates (Samuni et al, 2021). Both species also show a high level of altruism among members of the same group; in fact, intragroup altruism and intergroup violence may have co-evolved in humans (Rusch, 2014). This raises the question whether altruism is linked to our inherent tendencies to attack other groups in an organized manner, that is, war.

Altruism as defined by the Merriam–Webster dictionary is the “unselfish regard for or devotion to the welfare of others”. It is an individual’s emotional readiness to devote or even sacrifice his or her own life for the “greater good” of the group of which he or she is part. This can encompass anything from a small number of people in a “tribe” up to millions of citizens of a nation or followers of the same faith. The number does not matter: it is a shared belief, a piece of land or a nation, or any other common purpose that unites humans into larger, organized groups. As a result, the individual’s sense of life and goals are deeply linked to the group’s purpose or beliefs.

## Symbols and roots

In his book *Sapiens*, the Israeli historian Yuval Noah Harari describes two phases in human history. From 200–300,000 to about 70,000 years ago, *Homo sapiens* was only one of various hominid species. The second phase, from 70,000 years ago until today coincides with the expansion of *Homo sapiens* at the detriment of others, including the disappearance of the Neanderthals, with whom Sapiens was intermingling and mating, around 30,000 years ago. What is the reason for Neanderthal’s demise and Sapiens’ success? Harari argues that it was the power of symbolic thinking and abstraction, with ‘images’, such as ancestry, nation and gods, that enabled our ancestors to organize in larger communities, eventually expanding to encompass cities, states and empires. Overpowering all these symbols, according to Harari, is a cross-border “reality”, another invention of the human mind: money. Indeed, among the countless symbols throughout human history, money could be regarded as the ultimate guiding principle that transcends cultural barriers. This echoes Max Weber’s *The Protestant Ethic and the Spirit of Capitalism* that explores the interplay between religion and economics with broad implications for sociological and economic history.

Towards the end of the Roman Empire, the emperors extensively used money to bribe enemies and to recruit people into the army, but that proved ultimately futile against the successive waves of invasions. Even money was not powerful enough to appease Germanic tribes bent on invasion. An inherent limit to the supremacy of money would be “*enracinement*” (*the need for roots)* as the French philosopher, mystic and political activist Simone Weil discussed in her book of the same title, first published in France in 1942. *Enracinement* describes the profound human need for roots, the existential necessity of belonging to a community that is firmly grounded in a shared past, in present reality and its future aspirations.

“*Enracinement* describes the profound human need for roots, the existential necessity of belonging to a community that is firmly grounded in a shared past, in present reality and its future aspirations.”

All forms of life, even the simplest bacteria, require the establishment of specific coordinates in space and time for orientation and direction. This holds true for individuals and groups, whether these are ant colonies, bird flocks, wolf packs, chimpanzee groups or human tribes. Philosophers often refer to this shared foundation as “Being”, which is not an abstract notion but a matter of life and death: by not being anchored to reality, there is the risk of losing orientation and direction and, ultimately, destruction and death. *Sein und Zeit* (Being and Time) is the title of Martin Heidegger’s most renowned book, written by the prominent philosopher while active in Nazi Germany. Heidegger emphasized the importance of being in the present moment (*da sein*), which transcends and surpasses any rational arguments.

However, discounting reason comes at a grave risk. Heidegger was not the first to ponder on existentialism: existential philosophy goes back millennia to a time when science and philosophy were not disjointed, when Greek thinkers used the power of reason to delve into the basic elements of the universe. From them came the insight that atoms, minuscule particles, are the basis of all realities. Pushing the exploration further and striving to understand the building blocks that hold human culture and society together, Greek philosophers explored the basis of what can be agreed to be good and evil, beautiful and ugly, right and false. Socrates, who dwelt on these questions, lived under the main principle that he knew only one thing: that he knew nothing. This simple principle, which still rings true in modern science as the Popperian principle of falsifiability, inspired him to walk the streets of Athens and establish a dialog with others to bring out common principles of human existence. Of note is that Socrates was sentenced to death for pursuing his unquenched thirst of truth without compromise.

## Totalitarianism

Heidegger’s thoughts on “being here and now” and his attack on the foundation of Western metaphysics have deeply inspired the work of Alexander Dugin, a Russian philosopher with close ties to Vladimir Putin. Dugin’s philosophy draws from Orthodox Christian values and mysticism, and rejects what he sees as the materialism and soullessness of the West. With his “fourth political theory”, he proposes an alternative to the three main 20th-century ideologies—liberalism, communism, and fascism—advocating a multipolar vision, with Eurasia—and thereby Russia—as a major pole. Dugin’s political theory, which is compatible with Russian imperialism, criticizes the Western focus on maximizing liberty for each individual and argues that the state should instead provide the needed freedom for its greatest figures to maximize their capacities (Millerman, 2023).

Dugin’s work is reminiscent of what is written in Hannah Arendt’s book *The Origins of Totalitarianism*. In the 13^th^ chapter, on Ideology and Terror, Arendt says that totalitarianism “operates neither without guidance of law nor is it arbitrary, for it claims to obey strictly and unequivocally those laws of Nature or of History from which all positive laws always have been supposed to spring […]. Far from wielding its power in the interest of one man, it (totalitarianism) is quite prepared to sacrifice everybody’s vital immediate interest to the execution of what it assumes to be the law of History or the law of Nature”.

When groups or nations assert their roots at the expense of others, believing that they abide to a law of history or nature, conflicts become an existential question that cannot be resolved or stopped by reasonable arguments or bribes as the Roman emperors did. While defense and the threat of a counterstrike are essential to deter a foe from attacking in the first place, they cannot overcome ideologies that pervert the deep-seated human need for roots into hatred of ‘the others’. Instead, faced with the threat of destruction, even democratic societies are tempted to adopt the same mindset as totalitarian regimes, dividing people into “good and bad”, “us versus them”.

… “faced with the threat of destruction, even democratic societies are tempted to adopt the same mindset as totalitarian regimes, dividing people into “good and bad”, “us versus them”.

## Common ground

Amidst the disastrous suffering and deaths of so many people, it is imperative to find common ground to build bridges and bring reason again to the forefront. Traditional approaches to answer questions of life and death, such as religion and philosophy can be inadequate, as they are easily manipulated and distorted. However, there is an undeniable and simple biological truth that overpowers any form of manipulation. We all started life in the womb of a woman, we all eat and sleep, and we all die.

Today’s medicine is firmly rooted in the unquestionable knowledge of these vital processes, orchestrated by molecules, cells, tissues and organs and the mechanisms that connect them. The enormous successes of the biomedical sciences are the result of the reductionistic and mechanistic thinking of the modern age and rigorous experimental research. Biological and medical knowledge and insights have also the immense power to remind us of how much we have in common. We are all members of the same species, our genomes are 99.9% identical to each other and the functioning of our brains, perception of pain and pleasure, are by and large the same. Going back to Weil’s book, now as relevant as ever (Zaretsky, 2019), biomedical sciences should remind all people of their basic needs, with those for food and shelter closely linked to that of common roots.

The causes and nature of warfare have evolved over time, shaped by human biology, culture, technology and social organization. Among them remains a lack of reason and critical thinking, as the absence of thought is what drives humans to kill each other. Arendt’s book *The banality of evil* finds a counterpart in her lecture on “*thinking and moral considerations*” (Arendt, 1971) as well as her final book on *The life of the Mind*. To overcome our innate tendency to kill each other in the name of a nation or a god, we instead need to establish a dialog about our differences but more importantly about our shared humanity and roots. But to practice a discourse based on reason and to bridge divides, larger settings such as virtual platforms, conferences or concert halls can be insufficient. Smaller gatherings have a better chance of bringing people together and rediscover the fundamental ties that unite all of humanity. In that way, people willing to meet the ‘other’ and question their own values can step outside the flawed logic of war to become ‘ambassadors of peace’.

“To overcome our innate tendency to kill each other in the name of a nation or a god, we instead need to establish a dialog about our differences but more importantly about our shared humanity and roots.”

## Supplementary information

Peer Review File

## References

[CR1] Arendt H (1971) Thinking and moral considerations: a lecture. Soc Res 38:417–446

[CR2] Daly M, Wilson M (1988) Homicide. Gruyter, New York

[CR3] Fry DP (2012) Life without war. Science 336:879–884 22605769 10.1126/science.1217987

[CR4] Glowacki L, Wilson ML, Wrangham RW (2020) The evolutionary anthropology of war. J Econ Behav Organ 178:963–982 10.1016/j.jebo.2017.09.014

[CR5] Meijer H (2024) Janus faced: the co‐evolution of war and peace in the human species. Evol Anthropol Issues News Rev 33:e22027 10.1002/evan.22027 38623594

[CR6] Millerman M (2023) Alexander Dugin Explained. First Things https://www.firstthings.com/article/2023/02/alexander-dugin-explained

[CR7] Raine A, Yang Y (2006) Neural foundations to moral reasoning and antisocial behaviour. Soc Cogn Affect Neurosci 1:203–13 18985107 10.1093/scan/nsl033 PMC2555414

[CR8] Rusch H (2014) The evolutionary interplay of intergroup conflict and altruism in humans: a review of parochial altruism theory and prospects for its extension. Proc R Soc B 281:20141539 25253457 10.1098/rspb.2014.1539 PMC4211448

[CR9] Samuni L, Crockford C, Wittig RM (2021) Group-level cooperation in chimpanzees is shaped by strong social ties. Nat Commun 12:539 33483482 10.1038/s41467-020-20709-9 PMC7822919

[CR10] Zaretsky R (2019) Rooting for a New Patriotism. Foreign Affairs, June 28, 2019. https://www.foreignaffairs.com/articles/france/2019-06-28/rooting-new-patriotism

