# Supplementary material for: Ambassadors of peace: The anthropology of war and how to overcome the human killing instinct
Source: EMBO Rep. 2024 Aug 19;25(9):3753–6. doi: 10.1038/s44319-024-00231-5 (PMC11387388; doi:10.1038/s44319-024-00231-5)
Supplement: Supplementary file 1 — Peer Review File [file 44319_2024_231_MOESM1_ESM.pdf]

EMBO reports

Ambassadors of peace: The anthropology of war and and how to overcome the human killing instinct

Gian Paolo Dotto

Corresponding author: Gian Paolo Dotto (paolo.dotto@unil.ch)

Review Timeline:

Submission Date:	9th Aug 24
Accepted:	9th Aug 24

Editor: Holger Breithaupt

Transaction Report: This article was editorially reviewed
